# Feasibility and Safety of Bilateral Hybrid EEG/EOG Brain/Neural–Machine Interaction

**DOI:** 10.3389/fnhum.2020.580105

**Published:** 2020-12-09

**Authors:** Marius Nann, Niels Peekhaus, Cornelius Angerhöfer, Surjo R. Soekadar

**Affiliations:** ^1^Clinical Neurotechnology Lab, Charité – University Medicine Berlin, Berlin, Germany; ^2^Applied Neurotechnology Lab, University Hospital Tübingen, Tübingen, Germany

**Keywords:** bilateral exoskeleton control, bimanual tasks, EEG, EOG, brain-computer interface, BCI, brain-machine (computer) interface

## Abstract

Cervical spinal cord injuries (SCIs) often lead to loss of motor function in both hands and legs, limiting autonomy and quality of life. While it was shown that unilateral hand function can be restored after SCI using a hybrid electroencephalography/electrooculography (EEG/EOG) brain/neural hand exoskeleton (B/NHE), it remained unclear whether such hybrid paradigm also could be used for operating two hand exoskeletons, e.g., in the context of bimanual tasks such as eating with fork and knife. To test whether EEG/EOG signals allow for fluent and reliable as well as safe and user-friendly bilateral B/NHE control, eight healthy participants (six females, mean age 24.1 ± 3.2 years) as well as four chronic tetraplegics (four males, mean age 51.8 ± 15.2 years) performed a complex sequence of EEG-controlled bilateral grasping and EOG-controlled releasing motions of two exoskeletons visually presented on a screen. A novel EOG command performed by prolonged horizontal eye movements (>1 s) to the left or right was introduced as a reliable switch to activate either the left or right exoskeleton. Fluent EEG control was defined as average “time to initialize” (TTI) grasping motions below 3 s. Reliable EEG control was assumed when classification accuracy exceeded 80%. Safety was defined as “time to stop” (TTS) all unintended grasping motions within 2 s. After the experiment, tetraplegics were asked to rate the user-friendliness of bilateral B/NHE control using Likert scales. Average TTI and accuracy of EEG-controlled operations ranged at 2.14 ± 0.66 s and 85.89 ± 15.81% across healthy participants and at 1.90 ± 0.97 s and 81.25 ± 16.99% across tetraplegics. Except for one tetraplegic, all participants met the safety requirements. With 88 ± 11% of the maximum achievable score, tetraplegics rated the control paradigm as user-friendly and reliable. These results suggest that hybrid EEG/EOG B/NHE control of two assistive devices is feasible and safe, paving the way to test this paradigm in larger clinical trials performing bimanual tasks in everyday life environments.

## Introduction

Cervical spinal cord injuries (SCIs) often result in loss of motor function in all four extremities. According to the National Spinal Cord Injury Statistical Center (NSCISC), 41.1% of all SCIs lead to complete or incomplete tetraplegia (National Spinal Cord Injury Statistical Center, 2019). While the inability to walk is usually sufficiently compensated by use of a wheelchair (Rushton et al., 2010), restoration of hand and arm function is still insufficiently solved. Therefore, restoration of hand and arm function is of highest priority in this patient population (Anderson, 2004; Snoek et al., 2004; Lo et al., 2016). Depending on the SCI’s location, the degree of impairment and related motor inabilities can vary substantially. In particular, injuries between the spinal motion sections C5 and C7 are characterized by some remaining motor function in the shoulder and arm but absence of movements in the wrist and fingers (Ahuja et al., 2017). For these cases, restoration of hand function would be an important goal to regain autonomy and to improve quality of life (Campbell et al., 1999).

To date, the most common methods for restoration of upper limb motor function are surgical interventions (Bunketorp-Käll et al., 2017). To a certain degree, upper limb reconstructive surgeries, such as tendon transfers or tenodesis (Bednar and Woodside, 2018), can restore arm and hand function in SCI. However, besides the risks associated with surgery, tendon transfer strongly depends on the availability and quality of tendons and muscles suitable for transfer. While tenodesis enables tetraplegics to passively grasp objects through extension of the wrist (termed tenodesis grasp), the resulting grasping force is often insufficient to perform basal activities of daily living (ADLs), e.g., lifting up a water bottle, zipping a jacket, or reliably holding cutlery for eating (Dunn et al., 2016).

As an alternative to surgical interventions, recent advancements in neurotechnology and robotics opened up new possibilities to restore hand and arm function after cervical SCI (Soekadar et al., 2016) or stroke (Soekadar et al., 2008, 2015a; Nann et al., 2020). It was shown that exoskeletons or functional electrical stimulation (FES) of paralyzed muscles can enhance grasping force and improve hand function in tetraplegics (Ragnarsson, 2008; Ho et al., 2014; Yun et al., 2017; Cappello et al., 2018). A very intuitive way to control such assistive devices can be achieved by using a brain–computer interface (BCI; Wolpaw et al., 2002; Collinger et al., 2013a). BCIs translate electric, magnetic, or metabolic brain activity, e.g., associated with motor imagery (MI) or the attempt to move the paralyzed fingers, into control signals of digital devices, e.g., a robotic arm (Hochberg et al., 2012; Collinger et al., 2013b), exoskeleton (Soekadar et al., 2016; Tang et al., 2016; Frolov et al., 2017; Benabid et al., 2019), or FES device (Osuagwu et al., 2016; Vidaurre et al., 2016). Besides providing assistance, it was shown that repeated BCI use following SCI can also trigger neural recovery (Donati et al., 2016). Several studies showed that BCI-controlled FES can restore hand movement (Bouton et al., 2016; Vidaurre et al., 2016; Ajiboye et al., 2017). However, it is noteworthy that persons with SCI can develop upper extremity spasticity (Holtz et al., 2017; Gohritz and Fridén, 2018). In such cases, effective restoration of hand function *via* FES may not be successful due to increased muscle tone and tendon contractures. In contrast, a BCI-controlled hand exoskeleton, which actively opens and closes the affected hand, can overcome such limitations and may, thus, be superior to BCI-controlled FES. Within the last years, several robotic devices have entered the commercial market including three exoskeletons that were specifically designed for SCI patients (Mekki et al., 2018). Although still rather cost-intensive, new 3D-printed designs may yield low-cost hand exoskeletons in the near future (Yoo et al., 2019).

The most common approach for non-invasive brain/neural control of an exoskeleton uses modulation of sensorimotor rhythms (SMRs, 8–12 Hz) quantified as event-related desynchronization (ERD; SMR-ERD; Pfurtscheller and da Silva, 1999; Soekadar et al., 2011). SMR-ERD modulations related to MI or attempted finger movements are most prominent over the hand knob area of the contralateral primary motor cortex. Using electroencephalography (EEG), the optimal position to record SMR-ERD is typically at electrode positions C3 or C4 (according to the international 10/20 system; Neuper et al., 2006). Recently, it was demonstrated that a SMR-based brain/neural hand exoskeleton (B/NHE) can fully restore unilateral hand function in tetraplegics in an everyday life environment, e.g., to eat and drink in an outside restaurant (Soekadar et al., 2016). To deal with the inherent low signal-to-noise ratio of EEG recordings in everyday life environments, a hybrid EEG/electrooculography (EEG/EOG) brain/neural–machine interaction (B/NMI) system has been successfully introduced (Soekadar et al., 2015b, 2016; Crea et al., 2018; Nann et al., 2020). To enhance BCI control in everyday life environments, maximal horizontal oculoversions (HOVs) assessed by EOG were integrated as an additional control signal to reduce false classifications (Witkowski et al., 2014; Soekadar et al., 2015b). While exoskeleton closing motions were controlled by SMR-ERD related to intended grasping movements, HOVs were translated into opening motions or veto commands to interrupt unintended closing motions.

To date, the majority of studies in clinical settings have mainly focused on the restoration of *unilateral* motor function (Alam et al., 2016; Carvalho et al., 2019; Coscia et al., 2019). Most ADLs, however, involve *bilateral* motor function, e.g., eating with fork and knife, opening a water bottle, or a bag of potato chips. While, for example, a unilateral B/NHE might be sufficient to restore bimanual ADLs in hemiplegic stroke patients, patients suffering from tetraplegia depend on mobilization of both hands and arms to execute bimanual tasks. Therefore, a reliable and safe control paradigm allowing intuitive operation of bilateral hand exoskeletons would be very desirable.

The goal of such a bilateral control paradigm is to reliably detect the user’s attempt to operate either the left or right exoskeleton, both exoskeletons simultaneously, or none of them. This results in a four-class classification problem. The simplest approach to deal with such a multiclass problem is to implement a single classifier that differentiates between left and right hemispheric SMR-ERD (Meng et al., 2016; León, 2017; Lotte et al., 2018). Although Meng et al. (2016) demonstrated that this kind of classification method is feasible in principle, it requires sufficient lateralization of SMR-ERD to C3 and C4. Given that chronic tetraplegics often do not show such lateralization (Osuagwu et al., 2016; Dahlberg et al., 2018), such approach may not be suitable for reliable exoskeleton control in SCI. A possible solution to overcome the lack of lateralization in SCI patients is to introduce a reliable switch to activate either the left or right exoskeleton.

Here, we introduce a novel EOG command performed by *prolonged* HOV (>1 s; Figure 3) to the left or right and tested whether use of such new command allows for reliable control of two hand exoskeletons. The prolonged HOV is not in conflict with the already established hybrid EEG/EOG paradigm according to Soekadar et al. (2016), where a *short* HOV (<1 s; Figure 3) is used to veto an ongoing exoskeleton opening or closing. To test the feasibility and safety of such novel bilateral EEG/EOG-based B/NMI control, eight healthy participants as well as four chronic tetraplegics performed a neurofeedback paradigm consisting of a complex sequence of bilateral grasping and releasing motions of two exoskeletons visually presented on a screen. In the following work, feasibility was defined as fluency and accuracy of bilateral EEG/EOG B/NHE control. While fluent control was defined as “time to initialize” (TTI) EEG-controlled operations in average below 3 s (i.e., valid SMR-ERDs were detected in average within 3 s; Crea et al., 2018), reliable control was defined as average classification accuracy above 80%, following the recommendation of Vidaurre and Blankertz (2010) and Ortner et al. (2015), e.g., when benchmarking common spatial patterns (CSPs). Safety requirements were met when all unintended closing motions were interrupted by using short HOV before the exoskeleton was fully closed. This means the “time to stop” (TTS) all unintended closing motions ranged within 2 s, the time of a full exoskeleton closing motion. Moreover, user-friendliness of bilateral control was assessed among tetraplegics by using a Likert scale.

## Materials and Methods

### Participants

Eight BCI-naive healthy participants (six females, mean age 24.1 ± 3.2 years) and four BCI-naive chronic tetraplegics (four males, mean age 51.8 ± 15.2 years, time since injury > 2 years) with complete [*n* = 2; American Spinal Injury Association (ASIA), grade A] and incomplete (*n* = 2, ASIA grades B and C) SCI (injury location between C5 and C7) were invited to a single-session experiment at the University Hospital of Tübingen, Germany. Before entering the study, all participants provided written informed consent. The study protocol complied with the Declaration of Helsinki and was approved by University of Tübingen’s local ethics committee (registration code of ethical approval: 201/2018BO1).

### Experimental Setup and Biosignal Online Processing

**FIGURE 1:**
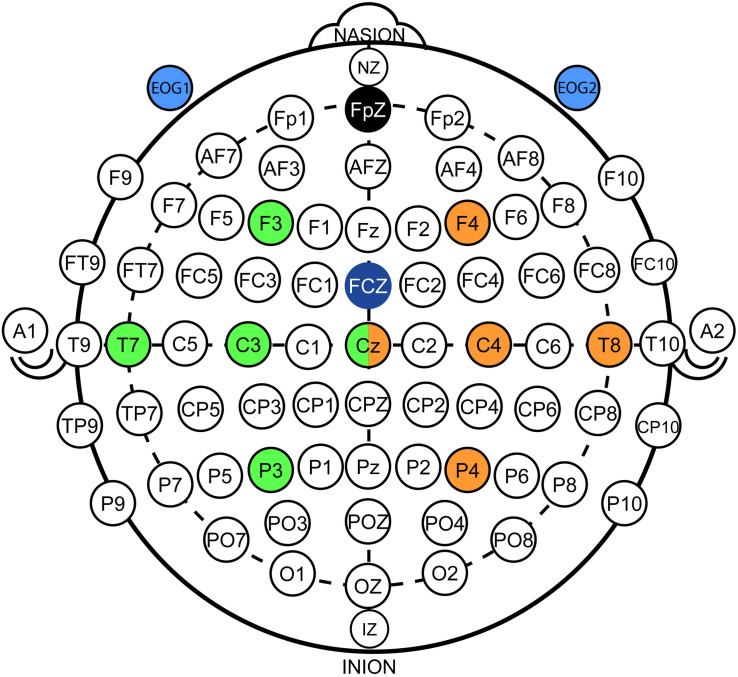
Electroencephalography/electrooculography (EEG/EOG) electrode setup. EEG setup: Nine conventional EEG recording sites were used in accordance to the international 10/20 system. Five electrodes on each hemisphere were applied that were centered around C3 (green color coding) and C4 (orange color coding). Signals from Cz were used for both hemispheres. EOG setup: Two EOG electrodes (light blue color coding) were placed laterally of the outer canthi of the left and right eye to assess horizontal oculoversions (HOVs) based on the bipolar EOG signal (i.e., difference between EOG1 and EOG2). Ground and reference electrodes were placed at Fpz (black color coding) and FCz (dark blue color coding), respectively.

**FIGURE 2:**
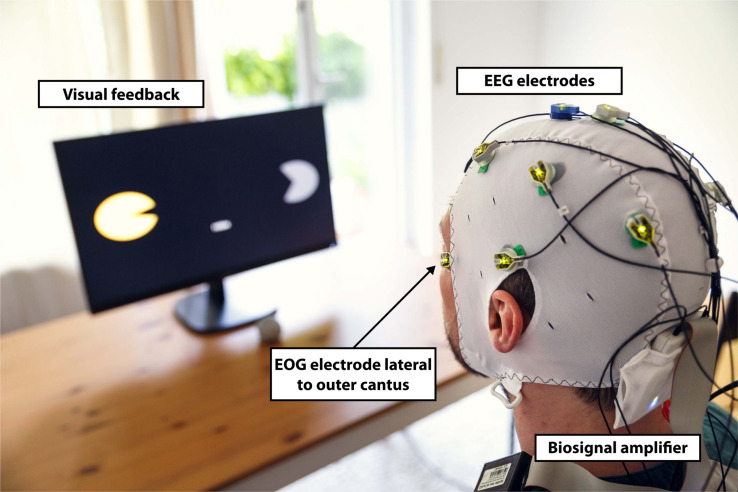
Experimental setup. Participants were equipped with a wireless active-electrode electroencephalography/electrooculography (EEG/EOG) recording system (actiCAP^®^, LiveAmp^®^, Brain Products GmbH, Gilching, Germany) and comfortably seated in front of a screen receiving visual feedback. Feedback included information about the task to be executed (in the middle of the screen) and the representation of the left (partly opened yellow circle indicates active exoskeleton) and right exoskeleton (partly opened gray circle indicates inactive exoskeleton) visualizing opening or closing motions. The figure shows an EOG electrode laterally placed to left outer cantus and five EEG electrodes arranged over the left hemisphere to assess Laplace-filtered brain activity at C3.

**FIGURE 3:**
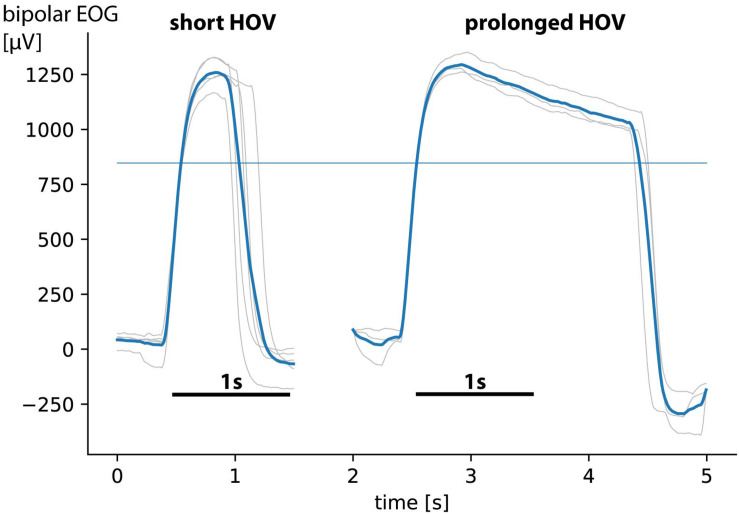
Short horizontal oculoversions (HOVs) vs. prolonged HOVs (figure shows only curve shape in positive direction resulting from left eye movements; curve shapes in negative direction from right eye movements are not visualized). Gray curve shapes show single trials; bold blue lines show average HOVs. Comparisons reveal distinct time difference between short HOV (<1 s) and prolonged HOV (>1 s, with its characteristic rectangular curve shape). The thin blue line indicates the 70% detection threshold.

Electroencephalography was recorded from nine conventional recording sites (F3, T3, C3, P3, F4, T4, C4, P4, and Cz according to the international 10/20 system; Figure 1). Two additional EOG electrodes were placed laterally to the outer canthi of the left and right eye to assess HOVs (Figures 1, 2; Heide et al., 1999). A reference electrode was symmetrically placed over the sagittal midline at FCz to avoid biased electrical potentials toward one hemisphere (Figure 1). The ground electrode was located at Fpz (Figure 1). All biosignals were sampled at 1 kHz and amplified by a wireless active-electrode EEG system (actiCAP^®^, LiveAmp^®^, Brain Products GmbH, Gilching, Germany; Figure 1). To ensure high signal quality, all impedances were kept below 25 kΩ. For online processing and classification, the BCI2000 software platform was used (Schalk et al., 2004). In order to attenuate eye blinks and other bihemispheric artifacts, bipolar EOG signal was calculated by subtracting left from right EOG. To remove low-frequency drifts as well as high-frequency noise, the bipolar EOG signal was then band-pass filtered with a first-order Butterworth filter at 0.02–3 Hz. To reduce the relatively long settling time that the low high-pass corner frequency at 0.02 Hz would have caused (>50 s), the band-pass filter was initialized with the mean value of the first processed sample block of the bipolar EOG signal. Such filter initialization drastically reduced the settling time to be applicable in online settings. The very low frequency content in the EOG signal allows to extract the quasi-rectangular curve shapes resulting from HOVs and thus ensures reliable detection of prolonged HOVs (i.e., threshold was exceeded for >1 s; Figure 3). EEG signals were first band-pass filtered with a first-order Butterworth filter at 1–30 Hz to remove baseline drifts and high-frequency noise. Afterward, surface Laplacian filters were applied to increase signal-to-noise ratio of the target electrodes at C3 and C4, respectively, (McFarland, 2015). A surface Laplacian filter was shown to be effective in detecting motor-specific SMR-ERD especially in online settings while suppressing distant sources (e.g., eye blinks) without the need for complex models, e.g., accounting for volume conduction. Subsequently, the power spectra of Laplace-filtered C3 and C4 EEG signals were estimated online from 500 ms moving windows based on an autoregressive model of order 100 (Burg algorithm; Soekadar et al., 2011). Dependent on the optimal SMR frequency showing the largest modulation between 8 and 13 Hz during motor imagination/attempted finger movements vs. rest, the accumulated power of a 3-Hz bin around that modulation frequency [frequency of interest (FOI) ± 1.5 Hz] was extracted. Lastly, SMR-ERD related to imagined or attempted right- or left-hand movements was computed according to the power method described by Pfurtscheller and Aranibar (1979):

(1) $$RV=\frac{1}{\left|T_{ref}\right|}\sum_{t\in T_{ref}}P_{t}$$

(2) $$ERD\left(t\right)=\frac{P_{t}-RV}{RV}\times 100\%$$

where *P*_*t*_ is the estimated power of the 3-Hz-wide bin at every sample block *t*. RV is the reference value to normalize power *P*_*t*_ to receive the instantaneous *ERD*(*t*) at every sample block *t*. Notably, to receive ERD related to Laplace-filtered C3 (C3-ERD) and C4 (C4-ERD) EEG signals, two identical SMR-ERD processing pipelines were implemented in parallel for online calculation.

### Brain–Computer Interface Calibration and Familiarization

To calibrate HOV detection thresholds for each side, participants were instructed to perform 5 *short* as well as 3 *prolonged* HOVs to each side, respectively. HOV detection thresholds were set at ±70% of median single-trial EOG maxima and minima (median was selected to receive a more robust estimation; Figure 3). To determine the C3- as well as C4-ERD detection thresholds, two calibration runs were conducted. During the first run, participants were instructed to either imagine (healthy participants)/attempt (tetraplegics) left or right finger movements (active phases) or to relax (rest phases) according to 20 externally paced randomized visual cues lasting 5 s each. After each active or rest phase, an intertrial interval (ITI) with a randomized length of 4–6 s followed. After the first run, FOI was set to the optimal SMR frequency, and RVs for C3 and C4 were determined as average power of the entire run including all active and rest phases as well as all ITIs. During the second run, which consisted the same 20 visual cues, participants received online visual feedback based on their elicited SMR-ERD at C3 and C4. Finally, individual SMR-ERD detection thresholds were set to the average C3- and C4-ERD elicited within all active phases, respectively. After successful calibration, several familiarization runs were performed until the participant felt comfortable with all control commands.

### Electroencephalography/Electrooculography-Based Bilateral Control Paradigm

**FIGURE 4:**
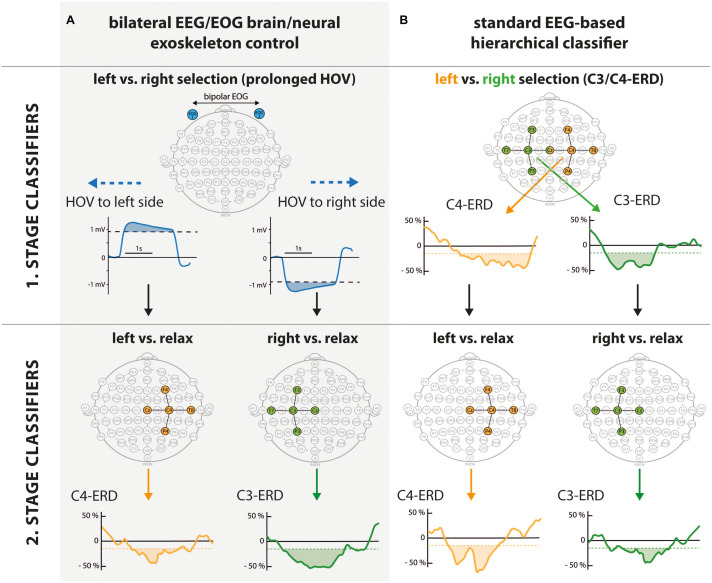
Hierarchical structure of bilateral electroencephalography/electrooculography (EEG/EOG) brain/neural exoskeleton control (**A**, gray shaded area) vs. standard EEG-based hierarchical classifier (**B**). While the user could select the left vs. right side at the first stage, closing vs. rest was classified at the second stage. **Comparison of first stage classifiers:** By using prolonged horizontal oculoversions (HOVs) to the left or right based on bipolar EOG (blue electrodes laterally placed to outer canthi), the subsequent classifiers at the second stage were activated. The solid blue line shows prolonged HOV signals exceeding the detection threshold for >1 s (blue shaded area). In contrast, a standard EEG-based hierarchical classifier requires distinct lateralization of event-related desynchronization (ERD) to C3 and C4. A common approach evaluates Laplace-filtered C3-ERD (green electrodes) and C4-ERD (orange electrodes) to classify the left vs. right side. To activate the left side (left branch), contralateral sensorimotor rhythm (SMR)-ERD at C4 (solid orange line) exceeding the C4-ERD detection threshold (orange shaded area) is needed. To select the right side, SMR-ERD at C3 (C3-ERD) needs to be detected accordingly (right branch). **Second stage classifiers:** At this stage, left/right vs. relax is distinguished. Depending on the classification at the first stage, electrodes of only one hemisphere are activated (green or orange electrode sites). Solid orange/green line shows valid C4-/C3-ERD (orange/green shaded areas). In case C4/C3-ERD detection thresholds were not exceeded, a relax state was detected. This classifier stage is identical for both approaches.

The EEG/EOG-based bilateral control paradigm was implemented as a hierarchical classifier with two sequential binary classification stages. This is a common approach to decompose the multiclass classification problem into several binary classification problems (Lotte et al., 2018). At the first stage, a linear classifier detected *prolonged* HOVs either to the left or to the right to activate the respective exoskeleton. As soon as the HOV detection threshold was exceeded for longer than 1 s, the classifier recognized this as a volitional laterality switch and enabled the specific classifier at the second stage. Dependent on the selected exoskeleton, either C3- or C4-ERD was then continuously analyzed and translated into closing motions as long as the laterality-specific ERD detection threshold was exceeded. The principle of this two-stage EEG/EOG-based hierarchical classifier is illustrated in Figure 4A. To open the closed exoskeleton or to interrupt (veto) an unintended closing motion, a *short* HOV to any direction reset the exoskeleton again. A *short* HOV was classified when HOV detection threshold was exceeded less than 1 s (see Figure 3 for differences in HOV type). Such hybrid *short* EOG/EEG-based paradigm was already successfully applied in tetraplegics during unilateral hand exoskeleton control (Soekadar et al., 2016). To ensure safety, *short* HOV commands had the highest priority to veto any ongoing action in case two EEG/EOG-based features were detected at the same time (see priority order in Table 1).

**TABLE 1:** Overview of brain/neural-machine interface (B/NMI) control commands.

*B/NMI control command*	*EEG/EOG-based feature*	*Respective task instruction with visual feedback*
Interrupt closing motion	*Short* HOV toward any side Example: Short HOV to any side to interrupt (veto) the left ongoing exoskeleton motion	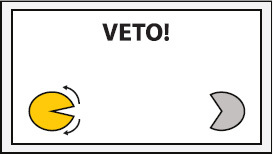
Open exoskeleton	*Short* HOV toward the direction of activated exoskeleton Example: *Short* HOV to the left to open left closed exoskeleton	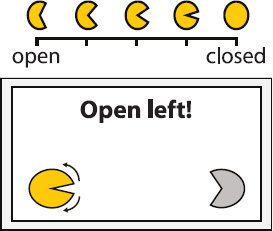
Switch active exoskeleton	*Prolonged* HOV (>1 s) toward desired hand exoskeleton Example: Before execute task instruction “Close left!,” *prolonged* HOV to the left is required to activate left exoskeleton	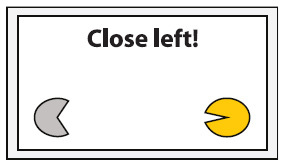
Close exoskeleton	SMR-ERD of contralateral motor cortex (C3- or C4-ERD) Example: C3-ERD required to close right exoskeleton	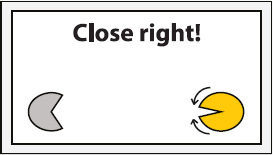
Rest	No action required.	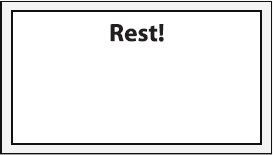

*The first column lists all possible commands for controlling each side. Importantly, the order of control commands listed in the table defines priority in case that two electroencephalography/electrooculography (EEG/EOG)-based features are detected at the same time starting with the highest priority at the top. The second column shows EEG/EOG-based features including examples for specific task instructions depicted in the third column. In the last column, visual feedback including exoskeleton motions for specific task instructions is illustrated. Yellow color coding indicates the active exoskeleton; gray color coding, the inactive exoskeleton. Only one possible instruction is illustrated for each control command. ERD, event-related desynchronization; HOV, horizontal oculoversion; SMR, sensorimotor rhythm.*

### Study Protocol and Audiovisual Online Feedback

To test for feasibility and safety of the novel EEG/EOG-based bilateral control paradigm, healthy participants as well as tetraplegics performed a pseudo-randomized sequence of 2 × approximately 40 subtasks consisting of all B/NMI control commands required for bimanual operation of the two visual exoskeletons (Table 1). The sequence included subtasks to close one of the exoskeletons (requiring C4- or C3-ERD), to open them again, or to stop (*veto*) an ongoing closing motion as fast as possible to simulate for unintended hand exoskeleton motions or unexpected incidents (the latter two required both *short* HOVs). In case a subtask required to close an exoskeleton, which had not been activated yet, participants first had to perform a *prolonged* HOV to the respective side before closing of the exoskeleton could be performed. To test for false positives, intervals to rest were randomly built in, in which the participants were instructed to avoid any action. A detailed overview on the bilateral B/NMI control commands, their corresponding EEG/EOG-based features, and their respective visual feedback are summarized in Table 1. To enhance reliable distinction of *short* vs. *prolonged* HOV, an auditory feedback with two different sounds was provided to confirm successful HOV execution. The time between subtasks varied randomly between 5 and 7 s. Each sequence lasted approximately 5 min. In case no SMR-ERD was elicited, subtasks were aborted after 10 s. The total number of HOV-based subtasks being executed slightly varied depending on the users’ previous SMR-ERD performance. For example, in case the user was not able to elicit ERD during a closing task, there was no need to reopen the exoskeleton again and was thus not requested. At the end of the session, tetraplegics rated user-friendliness of B/NMI control by using a five-level Likert-scale questionnaire. To account for the special needs of the tetraplegics, study protocols slightly differed between healthy participants and the patients. To reduce the overall session length, only six instead of eight rest phases were included. Moreover, the veto instructions were not randomly interspersed within the main study protocol but evaluated in a preceding pure EOG-based sequence. This was done to not overstrain the capabilities of the tetraplegic participants, since it was just a one-session study without any additional training day.

### Outcome Measures and Offline Data Analysis

Feasibility and safety of the novel EEG/EOG-based control paradigm were assessed according to the following outcome measures. Feasibility was defined as *fluency* and *accuracy* of EEG-controlled operations. Fluency of control was evaluated as time from appearance of task instruction until exceedance of the SMR-ERD detection threshold. In case a laterality switch was required, timer count started just after successful activation of the exoskeleton (by performing a *prolonged* HOV). Fluent control was assumed when the average TTI such EEG-controlled operations ranged below 3 s (Crea et al., 2018). To assess the accuracy of bilateral control, the two-stage classifier performance was evaluated. At the first stage, exoskeleton selection was considered valid when successful *prolonged* HOV was performed. At the second stage, a trial was counted as successful when a full exoskeleton closing motion was conducted requiring the side-specific SMR-ERD detection threshold to be exceeded by a minimum of 2 s in total. Accurate bilateral control was assumed when the accuracy of all classifiers exceeded 80% in average. Due to the fact that the sequence can contain different numbers of subtasks, the balanced accuracy was applied to account for a potential bias toward the more frequent class (Brodersen et al., 2010). The balanced accuracy is given by $\frac{1}{2}\left(\frac{TP}{P}+\frac{TN}{N}\right)$ weighting the true-positive and true-negative rate equally. Since classification stages were built up as binary classifiers, chance level ranged at 50%. To compare the presented hybrid EEG/EOG-based classifier accuracy with an implementation, which was built up with EEG-based binary classifiers only, an offline data analysis was performed. The different implementation methods at the first stage are illustrated in Figure 4. Unlike the online implementation, in which prolonged HOV (first stage) and side-specific ERD (second stage) were used, offline classification was only based on the recorded side-specific ERD (second stage of online paradigm) for both stages, since this was the classification while imagined/attempted finger movements were performed. This allowed comparison of the two approaches without the need to conduct two separate online sessions. Consequently, side-specific C3- and C4-ERDs were both classified depending on the instructed task. In case a left side closing was instructed, closing motions >2 s of the right or both exoskeletons or no movement were classified as false-negative events, whereas closing motion >2 s of the left exoskeleton was classified as a true-positive event. For the instruction to close the right side, the opposite events were classified: Movement of the right exoskeleton was classified as a true-negative event, while all other events were considered as false positives. To test for differences in average classification accuracy, a mixed-design analysis with “group” (healthy participants, tetraplegics) as between-group variable and “classification approach” (hybrid EEG/EOG brain/neural control, standard EEG-based hierarchical classifier) as repeated-measures variable was performed. To account for the limited number of data samples, bootstrapping was applied (Wilcox, 2011). Significance level was defined at *p* < 0.05. Safety was assumed when the TTS an unintended closing motion was interrupted within 2 s, meaning that closing motions were aborted before the exoskeleton was fully closed. Moreover, user-friendliness was met when the majority of tetraplegics rated EEG/EOG-based bilateral control as comfortable and easy to apply.

## Results

### Feasibility

**FIGURE 5:**
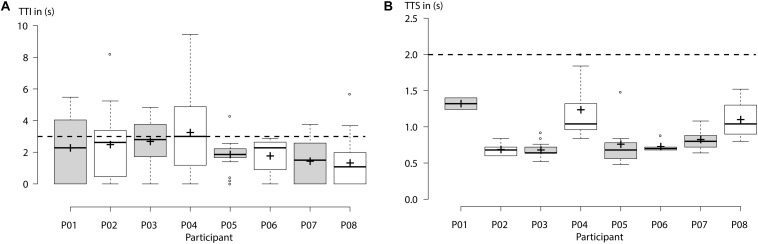
Healthy participants: **(A)** “Time to initialize” (TTI) electroencephalography (EEG)-controlled closing motions of the left- or right-hand exoskeleton for each participant. Horizontal dashed line indicates the threshold for fluency criterion set at 3 s. Average TTI across all subjects ranged below 3 s, documenting fluent control. **(B)** “Time to stop” (TTS) an ongoing closing motion by using *short* horizontal oculoversions (HOVs). Horizontal dashed line indicates the threshold for safety criterion set at 2 s. Centerlines of boxplot show the median, while crosses show the mean. Box limits indicate the 25th and 75th percentiles.

**FIGURE 6:**
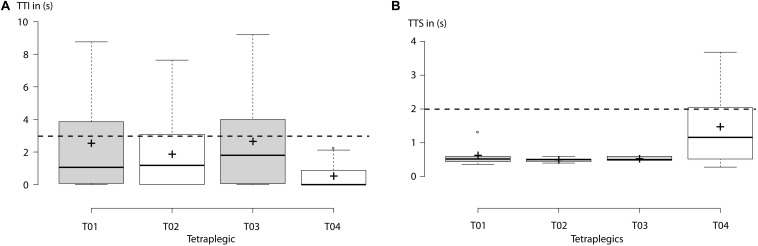
Tetraplegics: **(A)** “Time to initialize” (TTI) electroencephalography (EEG)-controlled closing motions of the left- or right-hand exoskeleton for each participant. Horizontal dashed line indicates the threshold for the fluency criterion set at 3 s. Average TTI across all subjects ranged below 3 s, documenting fluent control. **(B)** “Time to stop” (TTS) an ongoing closing motion by using *short* horizontal oculoversions (HOVs). Horizontal dashed line indicates the threshold for the safety criterion set at 2 s. Only tetraplegic T04 exceeded the threshold of safety criterion. Centerlines of boxplot show the median, while crosses show the mean. Box limits indicate the 25th and 75th percentiles.

Average TTI [mean TTI ± standard deviation (SD)] all EEG-controlled visual closing motions ranged at 2.14 ± 0.66 s across healthy participants and at 1.90 ± 0.97 s across tetraplegics, documenting *fluent* bilateral B/NMI control. Figures 5A, 6A show the individual TTI distribution for each participant. Only one healthy participant exceeded the fluency criterion (P04: 3.25 ± 2.65 s).

Average accuracy (mean ± SD) for bilateral EEG/EOG brain/neural exoskeleton control ranged across all classifiers (i.e., including 1. stage classifier: prolonged HOV, and 2. stage classifier: C3-/C4-ERD) at 85.89 ± 9.47% across healthy participants and at 81.25 ± 5.84% across tetraplegics (Figure 4A). For the standard EEG-based hierarchical classifier, average accuracy declined across all classifiers to 71.33 ± 17.21% among healthy participants and to 58.68 ± 10.62% among tetraplegics (Figure 4B). There was a significant main effect of “classification approach” (Ψ = −17.23, *p* < 0.001), confirming superiority of the novel bilateral EEG/EOG brain/neural control for both healthy participants as well as tetraplegics. There was no main effect of “group” (Ψ = 6.04, *p* = 0.419) and no interaction between “classification approach” and “group” (Ψ = 4.88, *p* = 0.449). Tables 2, 3 list individual accuracy rates for each healthy participant and tetraplegic as well as present accuracy results of all classifiers at every hierarchical classification stage. Chance level of binary classifiers ranged at 50%. Importantly, due to the novel implementation (compare Figure 4A), *prolonged* HOVs to activate either the right or left exoskeleton at the first stage were classified in 100% of the cases.

**TABLE 2:** Accuracy of bilateral electroencephalography/electrooculography (EEG/EOG) brain/neural exoskeleton control.

	Healthy participants				Tetraplegics			
	1. stage	2. stage		Total	1. stage	2. stage		Total
	Left/Right	Left/Rest	Right/Rest		Left/Right	Left/Rest	Right/Rest	
**No.**								
1	100.00	65.60	67.90	77.83	100.00	58.30	66.70	75.00
2	100.00	78.40	74.00	84.13	100.00	70.80	75.00	81.93
3	100.00	65.20	80.00	81.73	100.00	83.30	54.20	79.17
4	100.00	42.00	68.30	70.10	100.00	91.70	75.00	88.90
5	100.00	89.70	91.90	93.87				
6	100.00	94.40	100.00	98.13				
7	100.00	96.90	87.50	94.80				
8	100.00	78.40	81.20	86.53				
**Mean**	**100.00**	**76.33**	**81.35**	**85.89**	**100.00**	**76.03**	**67.73**	**81.25**
**SD**	**0.00**	**18.34**	**11.36**	**9.47**	**0.00**	**14.61**	**9.83**	**5.84**

*Mean values with standard deviation (SD) are provided in bold.*

**TABLE 3:** Accuracy of standard electroencephalography (EEG)-based hierarchical classifier.

	Healthy participants				Tetraplegics			
	1. stage	2. stage		Total	1. stage	2. stage		Total
	Left/Right	Left/Rest	Right/Rest		Left/Right	Left/Rest	Right/Rest	
**No.**								
1	59.80	53.10	55.40	56.10	33.30	50.00	45.80	43.03
2	73.20	69.00	74.00	72.07	54.20	66.70	66.70	62.53
3	75.70	65.20	80.00	73.63	54.20	83.30	50.00	62.50
4	42.20	38.90	68.30	49.80	62.50	75.00	62.50	66.67
5	92.90	89.70	91.90	91.50				
6	83.30	94.40	100.00	92.57				
7	77.30	96.90	75.00	83.07				
8	27.30	59.70	68.80	51.93				
**Mean**	**66.46**	**70.86**	**76.68**	**71.33**	**51.05**	**68.75**	**56.25**	**58.68**
**SD**	**22.03**	**20.99**	**14.05**	**17.21**	**12.46**	**14.22**	**9.94**	**10.62**

*Mean values with standard deviation (SD) are provided in bold.*

### Safety

Average TTS (mean TTS ± SD) ongoing closing motions using *short* HOVs ranged at 0.92 ± 0.26 s across healthy participants and at 0.78 ± 0.46 s across tetraplegics. Figures 5B, 6B show the individual TTS distribution for each participant. Only one tetraplegic did not meet safety requirements while requiring more than 2 s to stop ongoing closing motions in some of the trials (T04: average TTS ± SD ranged at 1.47 ± 1.24 s; Figure 6B).

### User-Friendliness

**FIGURE 7:**
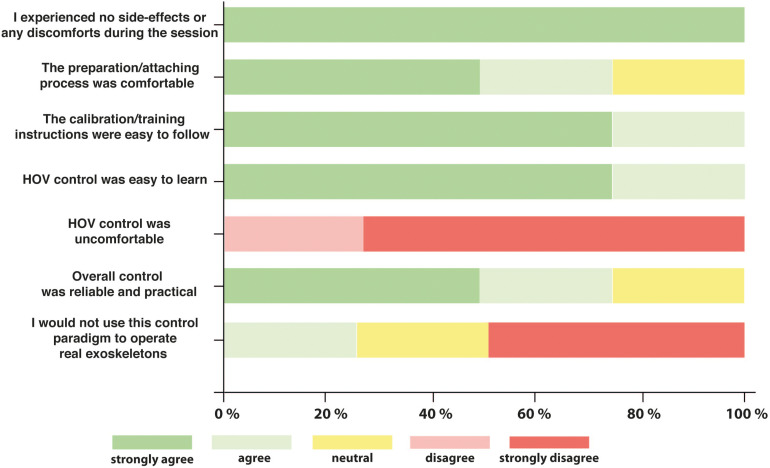
Five-level Likert scale questionnaire. After the experiment, all tetraplegics rated the user-friendliness of the overall process and especially the novel electrooculography (EOG) control commands. Likert scale ranged from 1 to 5 with “1 = strongly agree” and “5 = strongly disagree.”

With 88 ± 11% (mean ± SD) of the maximum achievable score, tetraplegics rated the novel bilateral EEG/EOG-based control paradigm as user-friendly and reliable. More specifically, all tetraplegics answered that they did not experience any side effects or discomfort, that the calibration/control instructions were easy to follow, and that the overall control was reliable and practical. Notably, all tetraplegics stated that the novel HOV-based control was easy to learn and that HOV control was comfortable. Importantly, three out of four tetraplegics would use the presented control to operate real hand exoskeletons bilaterally (Figure 7).

## Discussion

The presented study demonstrates feasibility and safety of a novel EEG/EOG-based B/NMI control paradigm for operating two hand exoskeletons. While feasibility was defined as fluency and accuracy of operation, safety was assumed when unintended closing motions could be aborted. We showed that eight healthy participants as well as four chronic tetraplegics were able to perform a complex sequence of subtasks mimicking bimanual tasks in daily life using four EEG/EOG-based control commands [i.e., side-specific SMR-ERD at C3 or C4, as well as *prolonged* (>1 s) and *short* HOVs; Table 1]. Fluent control was documented by an average TTI EEG-controlled operations below 3 s (2.14 ± 0.66 s across healthy participants and 1.90 ± 0.97 s across tetraplegics). These results are comparable to those of previous studies, in which a unilateral whole-arm exoskeleton was controlled by healthy participants (Crea et al., 2018) or stroke survivors (Nann et al., 2020). Accurate control was confirmed by an average classification accuracy exceeding 80% (85.89 ± 15.81% across healthy participants and 81.25 ± 16.99% across tetraplegics). Except for one tetraplegic, the TTS all ongoing motions were below 2 s (in average 0.92 ± 0.26 s across healthy participants and 0.78 ± 0.46 s across tetraplegics) underlining the system’s safety. Finally, user-friendliness among tetraplegics was proven by stating no discomfort and ease of use in controlling the B/NMI system for bilateral operation with 88 ± 11% of the maximal achievable scores.

These results demonstrate for the first time that the presented hybrid EEG/EOG-based B/NMI control paradigm can be used for reliable and safe operation of two hand exoskeletons, e.g., to perform bimanual tasks.

Control of two exoskeletons requires classification of more than two classes (multiclass classification). This problem can be solved either by directly applying multiclass methods, such as naive Bayesian classifiers (Suk and Lee, 2012; Zhang et al., 2015) or multilayer perceptrons (Balakrishnan and Puthusserypady, 2005), or, as more commonly used, by decomposing the problem into several binary classifications (Lotte et al., 2018). There are different possible decomposition methods, e.g., pairwise classification (Vuckovic et al., 2018) or by hierarchical classification (Dong et al., 2017; Gundelakh et al., 2018). However, all studies have relatively low binary classification accuracies in common ranging from 50 to 70%. To achieve a higher control accuracy, fusion of EOG- and EEG-based features was suggested and implemented in the presented bilateral control paradigm. A decisive step was to use a highly reliable EOG-based feature at safety-critical positions in the hierarchical classifier structure (Figure 4).

Fusing EEG with other biosignals like EOG is a well-established approach in the BCI field (Pfurtscheller et al., 2010). Soekadar et al. (2016) showed that such a hybrid EEG/EOG-based B/NHE fully restored hand function after SCI. Tetraplegics could eat and drink in a noisy outside restaurant by opening up the exoskeleton with *short* HOVs. This principle was now extended toward bilateral hand exoskeleton control introducing *prolonged* HOV. The advantage of this implementation was shown in the comparative offline analysis, where classification accuracy declined by 14.6% in healthy participants and by 22.6% in tetraplegics. The substantial decline in classification accuracy in tetraplegics compared to healthy participants underlines the need to compensate for the lack of lateralization in SCI by a reliable EOG-based switch between the two actuators.

One healthy participant (P04) did not meet the fluency criterion by 0.25 s in average, and one tetraplegic (T04) exceeded the safety criterion in some of the trials. However, in both cases, the unusually large SDs of 2.65 s for P04 and 1.24 s of T04 indicate that either the calibration threshold was not optimal or the participant did not attend to the task. Moreover, T04 was the only participant who stated that he would not want to use this paradigm in real life underpinning the previous assumptions.

Since EEG-based B/NMI control is generally more effortful than using other biosignals, e.g., electromyography (EMG) or HOV, one could argue that all exoskeleton movements could be controlled by HOV. However, contrary to eye movements, EEG-based control was shown to be more intuitive since exoskeleton closing motions are directly linked to imagining or attempting to move the paralyzed fingers (Soekadar et al., 2016).

Moreover, there is increasing evidence that repeated brain/neural control of exoskeletons can trigger neural recovery (Donati et al., 2016; Wagner et al., 2018). Therefore, a combination of both operational purposes, i.e., *assistive* and *restorative* use, was suggested (Soekadar et al., 2019; Soekadar and Nann, 2020). Here, the assistive neural exoskeleton is used as a technical aid for the physiotherapist to train the patient in performing ADLs. This hybrid approach promises to facilitate generalization of learned skills to real-life environments and may increase the impact of the rehabilitation treatment. The proposed B/NMI control paradigm paves the way toward implementation of such hybrid approach for restoration of bimanual ADLs.

Besides extending the existing EEG/EOG B/NMI control paradigm toward bilateral hand exoskeleton control, minimizing electrode biosignal recording sites constitutes another important step for everyday life applicability (Cavallo et al., 2020). Moreover, considering that the high classification accuracy (>80%) was achieved with a minimalistic setup of only nine EEG recording sites, this opens up new opportunities for an easy applicable EEG headset system without the need for time-consuming whole-head EEG recordings, which is usually needed for advanced CSP algorithms, achieving comparable classification results.

To reliably detect prolonged HOVs (>1 s), bipolar EOG signals have to contain low-frequency information. Therefore, a high-pass filter (lower cutoff frequency at 0.02 Hz) has to be used. As low-frequency bands are prone to be susceptible to movement artifacts, e.g., related to head movements, it needs to be tested whether the proposed approach for bilateral brain/neural exoskeleton control can be applied under less controlled and very noisy conditions (e.g., in an outside restaurant). Here, using other EOG signal features that are less dependent on information in the lower frequency bands could overcome this issue.

Larger clinical studies are needed to investigate whether these results can be generalized toward a broader spectrum of SCI patients. While all participants rated the brain/neural control paradigm as fluent, further increasing fluency would be desirable. In this context, taking advantage of lateralized brain activity [e.g., in the form of lateralized potential shifts preceding voluntary movements, the so-called Bereitschaftspotential or BP (Nann et al., 2019), or movement-related cortical potentials (MRCPs; Schwarz et al., 2020)] may contribute toward such aim. Since it was shown that SMR-ERDs are more pronounced over the contralateral hemisphere (Nikulin et al., 2008), it might be possible using advanced signal-processing tools to determine the side of the intended movement by assessing such lateralized activity only.

## Data Availability Statement

The raw data supporting the conclusions of this article will be made available by the authors, without undue reservation.

## Ethics Statement

The studies involving human participants were reviewed and approved by the Ethics Commission at the Medical Faculty of the Eberhard Karls University and the University Hospital Tübingen. The patients/participants provided their written informed consent to participate in this study.

## Author Contributions

MN and NP designed the study. NP collected the data of the healthy participants. MN, NP, and CA collected the data of the tetraplegics. MN and NP analyzed the data. MN, NP, CA, and SS interpreted the data and performed the literature search. MN, NP, CA, and SS wrote the manuscript. All authors contributed to the article and approved the submitted version.

## Conflict of Interest

The authors declare that the research was conducted in the absence of any commercial or financial relationships that could be construed as a potential conflict of interest.
